# Supplementary figures and images for: A Quantitative System for Discriminating Induced Pluripotent Stem Cells, Embryonic Stem Cells and Somatic Cells
Source: PLoS One. 2013 Feb 13;8(2):e56095. doi: 10.1371/journal.pone.0056095 (PMC3572019; doi:10.1371/journal.pone.0056095)

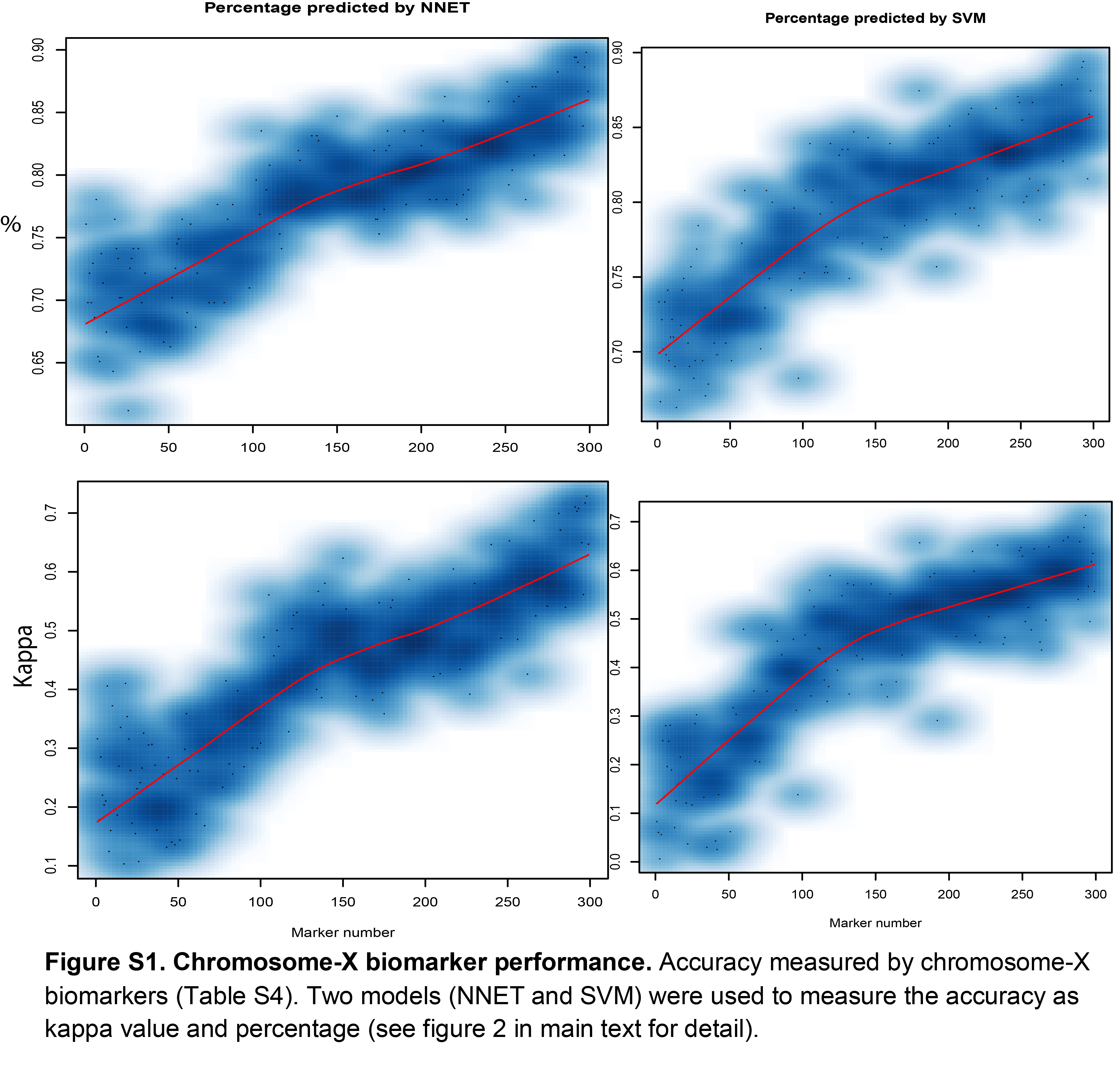

Supplement: Figure S1 — Chromosome-X biomarker performance. Accuracy measured by chromosome-X biomarkers (Table S4). Two models (NNET and SVM) were used to measure the accuracy as kappa value and percentage (see figure 2 in main text for detail). (TIF) [file pone.0056095.s007.tif]
